# Bacterial-Artificial-Chromosome-Based Genome Editing Methods and the Applications in Herpesvirus Research

**DOI:** 10.3390/microorganisms11030589

**Published:** 2023-02-26

**Authors:** Mengling Hao, Jiabao Tang, Shengxiang Ge, Tingdong Li, Ningshao Xia

**Affiliations:** 1 State Key Laboratory of Molecular Vaccinology and Molecular Diagnostics, Department of Laboratory Medicine, School of Public Health, Xiamen University, Xiamen 361102, China; 2 Xiang An Biomedicine Laboratory, Xiamen 361102, China; 3 NMPA Key Laboratory for Research and Evaluation of Infectious Disease Diagnostic Technology, School of Public Health, Xiamen University, Xiamen 361102, China; 4 The Research Unit of Frontier Technology of Structural Vaccinology of Chinese Academy of Medical Sciences, Xiamen 361102, China

**Keywords:** herpesvirus, bacterial artificial chromosomes, gene editing, virus mutant selection, reverse genetics, infectious clone

## Abstract

Herpesviruses are major pathogens that infect humans and animals. Manipulating the large genome is critical for exploring the function of specific genes and studying the pathogenesis of herpesviruses and developing novel anti-viral vaccines and therapeutics. Bacterial artificial chromosome (BAC) technology significantly advanced the capacity of herpesviruses researchers to manipulate the virus genomes. In the past years, advancements in BAC-based genome manipulating and screening strategies of recombinant BACs have been achieved, which has promoted the study of the herpes virus. This review summarizes the advances in BAC-based gene editing technology and selection strategies. The merits and drawbacks of BAC-based herpesvirus genome editing methods and the application of BAC-based genome manipulation in viral research are also discussed. This review provides references relevant for researchers in selecting gene editing methods in herpes virus research. Despite the achievements in the genome manipulation of the herpes viruses, the efficiency of BAC-based genome manipulation is still not satisfactory. This review also highlights the need for developing more efficient genome-manipulating methods for herpes viruses.

## 1. Introduction

Genome manipulation is an effective method for studying the function of viral genes and can help scientists understand the biology of viruses, such as discovering virulence factors and exploring new targets for developing novel vaccines and antiviral drugs for the prevention and treatment of viral infection. Herpesviruses are a group of double-strand DNA (dsDNA) viruses which can cause lifelong persistent infections and are major pathogens in humans and a wide range of animals. The genomes of herpesviruses are between 125 and 295 kilobases in length [1], which makes it difficult for genome manipulation.

In the past decades, scientists have developed several methods for manipulating the genome of herpesviruses, such as λ-red, Cre-*loxp*, CRISPR-Cas9, and others, which helped understand the pathogenesis of herpesviruses and develop novel vaccines and antiviral drugs [2,3]. Traditionally, linear DNA fragments or circular plasmids containing selection cassettes and flanking homologous regions were transferred into herpesvirus-infected cells, and the genomes of herpesviruses were edited by recombination, and then the recombinant viruses were selected by using different markers (different selection cassettes) [4,5,6,7]. This approach has a low efficiency. In addition, its dependence on the production of progeny viruses results in a limited range of genes that can be edited. Moreover, the cumbersome screening process for identifying functional recombinant viruses restricts its application [3]. Therefore, it is imperative to explore alternative techniques that can enhance the efficiency and expand the range of genes that can be edited. Bacterial artificial chromosomes (BAC), constructed based on factor F of *Escherichia coli* (*E. coli*), provide an important technical platform for the editing of large genomes, including herpesviruses [8,9,10,11]. BAC-based gene editing strategies are independent of the production of the progeny, and the screening efficiency is higher than conventional strategies [12,13,14]. Firstly, BACs can accommodate the insertion and stable inheritance of exogenous gene fragments ranging from 100 to 300 kb. The F factor facilitates the maintenance of low copy numbers (1–2 copies per cell) in *E. coli* which greatly reduces the difficulty in screening. Meanwhile, the development of various gene editing techniques in *E. coli* has greatly enhanced the editing efficiency of herpesvirus BACs compared to traditional methods. Edited BACs can be reintroduced into eukaryotic cells to reconstruct herpesvirus mutants for further study of their biological behaviors in cells. In addition, the selection of recombinantly engineered viruses is not dependent on the generation of progeny viruses, allowing editing of the *γ* herpes virus genome that maintains latent infection in the long term [13,15] as well as manipulating genes essential for the production of progeny viruses [11].

In this review, we will summarize the BAC-based herpesvirus genome editing techniques and selection methods and analyze the merits and drawbacks of different techniques. We will also summarize the applications of BAC-based genome editing techniques in herpesvirus research. This review could provide references for researchers to choose proper methods for manipulating herpesvirus genomes and assist the researchers in developing more efficient genome editing methods for herpes viruses.

## 2. Genome Editing Techniques of Herpesviruses Based on BAC

### 2.1. RecA Recombination Technique

**Figure 1:**
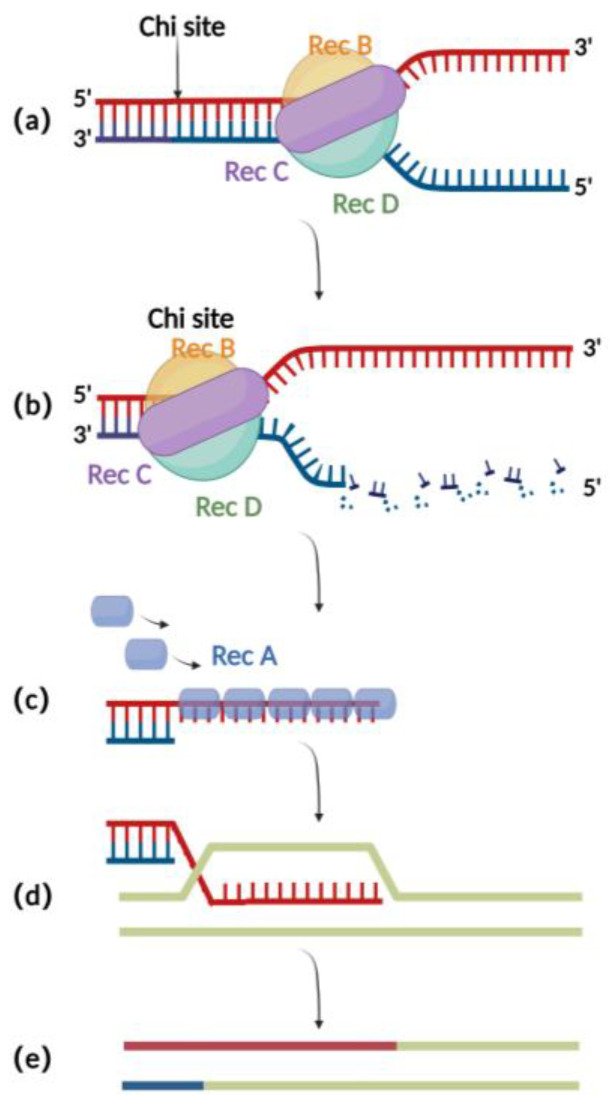
Schematic diagram of RecA recombination technique: (**a**) RecBCD binds to the double-stranded DNA terminus and promotes unwinding. (**b**) At the chi site, a single-stranded DNA is produced at the 3′ end. (**c**) The RecA protein binds to single-stranded DNA at the 3′ end. (**d**) ssDNA-RecA invades intact homologous double-stranded DNA for strand exchange. (**e**) Recombination completed.

The RecA recombination technique is a bacterial endogenous homologous recombination system composed of RecA recombinase and related auxiliary proteins (such as RecBCD, RecFOR, RuvABC, etc.). RecBCD is a multifunctional enzyme complex consisting of RecB, RecC, and RecD. When binding to the incision on the double-stranded donor DNA, RecB and RecD exhibit 3′ to 5′ and 5′ to 3′ helicase activities, respectively, which together result in the opening of the complementary double-stranded DNA (Figure 1a) [16]. Then, RecC can mediate the recognition of chi sites in the sequence and thereby cause allosteric changes, leading to a decrease in the pace of movement (Figure 1b) [17]. At this point, RecB also has exonuclease activity, which mediates the generation of single-stranded DNA at the 3′ end (Figure 1c) [18,19,20]. The RecA recombinase binds to single-stranded DNA sites generated by the exonucleases and engages in the process of identifying homologous regions on the BAC for subsequent annealing and interaction. After successful interaction, the nucleoprotein filaments invade the dsDNA and undergo strand exchange to form heteroduplex DNA (Figure 1d). Finally, RuvABC assists in catalyzing branch migration and degradation of the heteroduplex DNA, resulting in homologous recombination (Figure 1e) [21].

Although the RecA-mediated homologous recombination method has shown improved efficiency(10^−6^ to 10^−4^) compared to traditional methods for editing the herpesvirus genomes in eukaryotic cells (Table 1) [22], it still has significant drawbacks. One major issue is that due to the presence of repeated sequences in the herpes virus genomes, the expression of RecA can lead to instability in the herpes virus’s BAC clone, which in turn can cause mutations in the viral genome (Table 1) [1]. In addition, 500 bp to 3 kb long homologous arms were typically used in RecA-mediated recombination (Table 1) [7,23,24], resulting in a cumbersome process for constructing shuttle plasmids required for recombination. These drawbacks limit the application of RecA-mediated recombination.

### 2.2. λ-Red Recombination Technique

**Figure 2:**
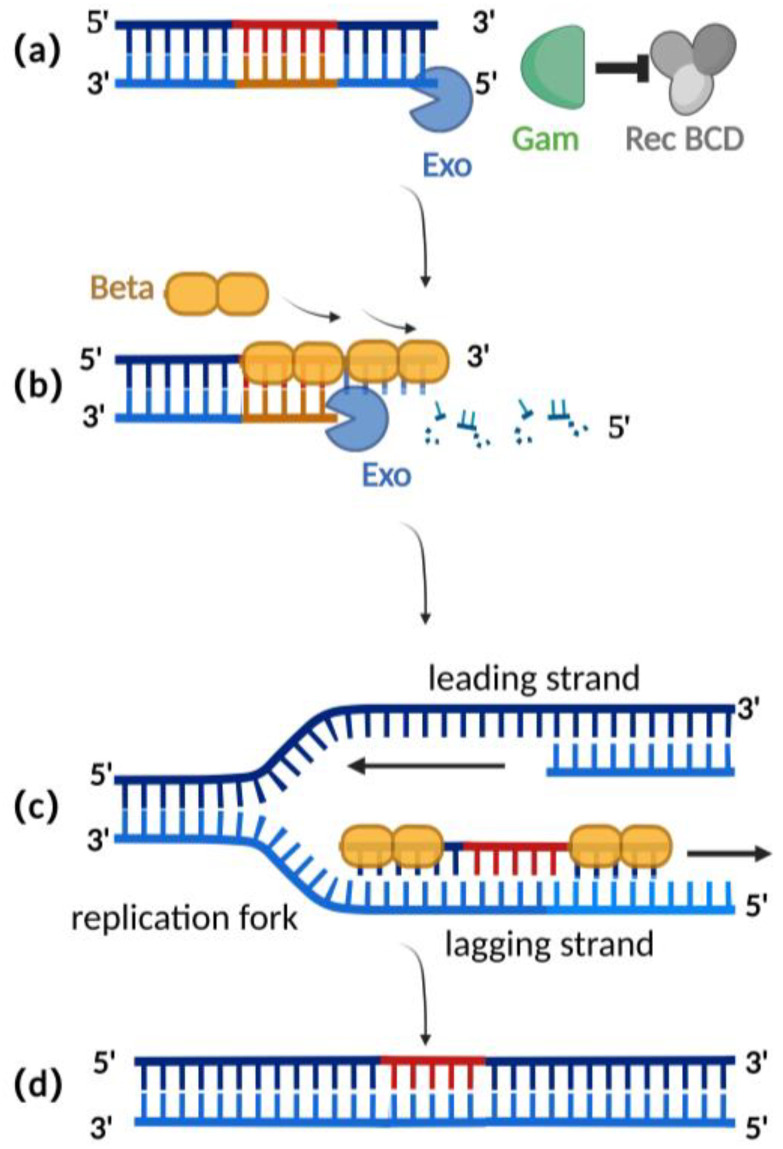
Schematic diagram of λ-red recombination technique: (**a**) Gam protein inhibits the activity of Rec BCD exonuclease; (**b**) Exo protein creates a stretch of single-stranded DNA at the 3′ end; (**c**) Beta proteins bind here, facilitating annealing interactions between the donor DNA fragment and the homologous sequence of the target site; (**d**) homologous recombination is complete with the replication of DNA.

Homologous recombination mediated by the λ-red recombination system is currently the most widely used recombination technique for editing herpesvirus BAC clones [2,25]. The λ-red recombination system is derived from the *phage λ* and consists of Gam, Exo, and Beta proteins. The Gam protein is an auxiliary protein of Exo protein and Beta protein. The Gam protein can inhibit the binding of Rec BCD to the ends of dsDNA and thus inhibit the function of Rec BCD exonuclease, preventing the degradation of the exogenous double-stranded DNA (Figure 2a) [26,27]. Exo proteins can bind to the ends of a dsDNA donor, which contains homologous fragments on both sides of the target gene. Concurrently, Exo protein possesses 5′ to 3′ exonuclease activity, producing a stretch of single-stranded DNA at the 3′ end (Figure 2b) [28]. Beta protein plays a decisive role in the process of λ-red homologous recombination. As a single-stranded DNA binding protein, the beta protein binds to the single-stranded DNA produced by the Exo protein. The binding of Beta protein enhances the annealing of the donor DNA fragment and the homologous sequence at the target site of the replicating herpes virus BAC (Figure 2c). Homologous recombination is complete with the replication of DNA (Figure 2d) [29,30].

Compared with the RecA recombination technique, the λ-red recombination technique avoids the risk of a partial deletion of the herpes virus genome in BAC during the recombination process as only homologous double-strand ends can be used as a substrate (Table 1). Additionally, this method typically only requires 30–50 base pairs of homologous arms for recombination (Table 1), making it easier to obtain the donor through techniques such as oligonucleotide synthesis or polymerase chain reaction (PCR), eliminating the need for shuttle plasmids as required in RecA recombination [31,32]. Importantly, the recombination efficiency mediated by the λ-red recombination technique has been improved to a maximum of 0.68% when the donor is double-stranded DNA (Table 1) [33,34,35].

### 2.3. Base Editing Technique

CRISPR/Cas 9 is a powerful genome editing technique [36] that has been successfully applied in a wide range of eukaryotic cells, including human cell lines, embryonic stem cells, *mice*, *Arabidopsis*, and *Drosophila* [37,38,39,40]. In 2013, Jiang et al. successfully edited *Streptococcus pneumonia*’s genome using CRISPR/Cas-9-only gene editing [41]. Since then, it has been successfully employed in a variety of prokaryotic species as well [42,43]. The versatility and effectiveness of CRISPR/Cas9 in modifying the genome make it a valuable tool for a wide range of scientific and medical applications.

Due to the lack of the non-homologous end-joining (NHEJ) pathway [44,45,46,47] and the low efficiency of their endogenous homologous recombination system, it is difficult to achieve stable genome editing in most bacteria using CRISPR/Cas9 gene editing technology alone [41,48,49,50]. Until now, there was still no publication reporting CRISPR/Cas 9-based gene editing technique in the stably preserved herpesvirus BAC gene in *E. coli.* This highlights the need to explore alternative approaches to achieve efficient and reliable gene editing in these organisms.

**Figure 3:**
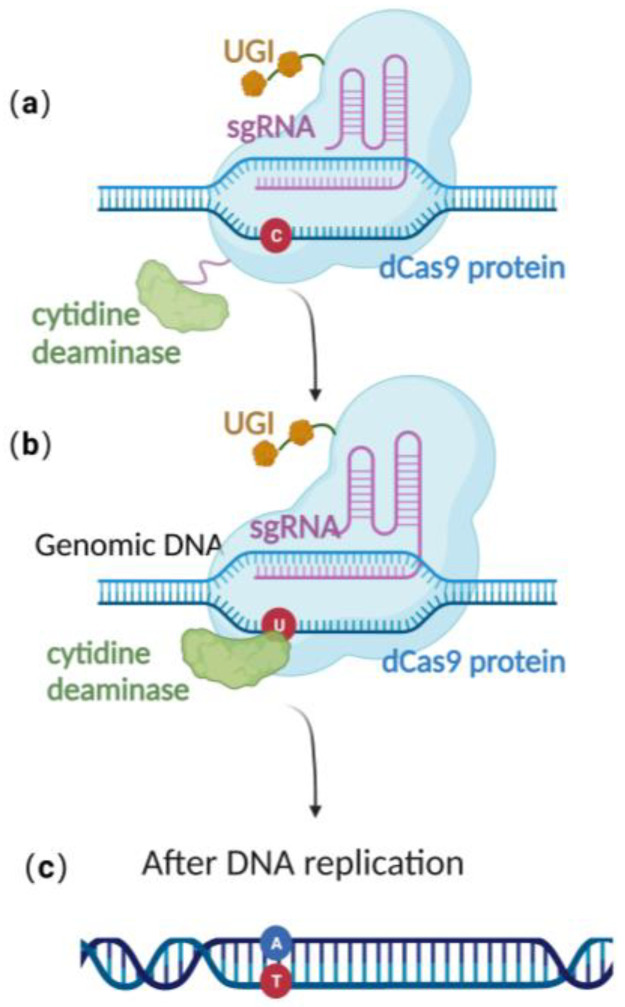
Schematic diagram of base editing method: (**a**) dCas9 mediates targeting without DSB formation; (**b**) cytosine deaminase converts C to U; (**c**) single-base precise editing is complete with the replication of DNA.

Recently, scientists have constructed an efficient gene editing method by combing CRISPR/Cas 9 gene editing technique with precise base editing technology. This method consists of an sgRNA and a complex that includes modified Cas9 proteins, cytosine deaminases, and an uracil glycosylase inhibitor (UGI) [51]. Unlike wild-type Cas9 proteins, the modified Cas9 is catalytically dead, lacking endonuclease activity. Therefore, it can only facilitate genome targeting via sgRNA but cannot induce a double strand break (DSB) due to the absence of cleavage activity (Figure 3a). Cytosine deaminase converts the specified cytosine (C) site to uracil (U) (Figure 3b). At this point, uracil glycosylase inhibitors can prevent the excision of intermediate product U, increasing the efficiency of converting C to T on the DNA chain, ultimately achieving single-base precise editing of C to T and G to A (Figure 3c).

Base editing allows for site-directed mutagenesis of multiple prokaryotic genomes [52], including *E. coli* [22] and even herpesvirus genome BACs preserved in *E. coli* [53]. Zheng et al. [22,53] utilized this technology, directly converting cytidine (C) to uridine (U) at specific positions on the US8 and UL34 genes of the pseudorabies virus genome BAC, thus achieving the premature termination of the corresponding genes and approaching 100% editing efficiency (Table 1). Due to the modifications that occur in the base-editing window, many studies have demonstrated that base editing is associated with off-target effects [54,55], thereby limiting its practical application. To effectively utilize base editing, optimization of the cytidine deaminase and/or UGI is necessary [56,57]. Despite this, the technology has the advantage of directly editing the target site nucleotide without the need for donors. It provides an efficient alternative method for point mutation editing.

Moreover, the novel combined editing technology that has emerged in bacterial genome editing but has not yet been applied to BACs and may also provide insights for future BAC editing. Combining CRISPR/Cas 9 with λ-red recombination could increase the efficiency of recombination in editing the genome of *E. coli*, with reported knockout efficiency of up to 100% for deletion lengths of up to 3.4 kb, which is higher than in previous reports [48,58,59]. The use of this system could potentially offer a new approach for efficient editing of the BAC of herpesvirus genomes that are stably stored in *E. coli*.

## 3. Screening Methods of Herpesvirus Mutants

**Figure 4:**
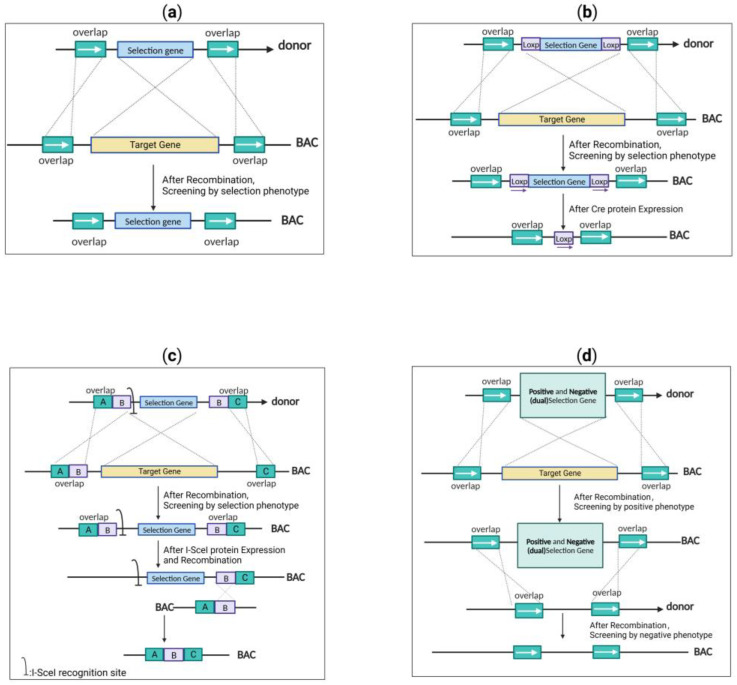
Schematic diagram of four different screening methods: (**a**) single selection cassette strategy (the phenotype of the selection cassette is screened after recombination, leaving the selection cassette); (**b**) selection cassette with site-specific recombinase recognition motif strategy (the phenotype of the selection cassette is screened in the first round of recombination). In the second round of recombination, the selection cassette is removed, leaving only the site-specific recombinase recognition motif by expressing the recombinase enzymes; (**c**) selection cassette with I-sceI endonuclease recognition site strategy (the phenotype of the selection cassette is screened in the first round of recombination). In the second round of recombination, the I-SceI site is cleaved and achieves scarless modification by expressing the I-sceI enzyme; (**d**) positive and negative (dual) selection cassette strategy (the phenotype of the positive selection condition is screened in the first round of recombination). In the second round of recombination, the phenotype of the negative selection condition is screened to achieve scarless modification.

Due to the insufficient efficiency of the abovementioned editing techniques, it was difficult to accurately identify and analyze any mutations that may occur during the editing process. Thus, a selectable marker was usually included in the donor gene for facilitating the selection of engineered clones (Figure 4). The design strategy of a suitable donor is essential to ensure the successful screening of virus mutants when editing the herpes virus genome. Donor designs are typically divided into four types: single selection cassette, selection cassette in combination with site-specific recombinase recognition motif, selection cassette in combination with I-sceI endonuclease recognition site, and positive/negative selection (dual-selection) gene.

### 3.1. Single Selection Cassette

For the single selection cassette strategy, in addition to homologous arms on both sides of the target site and the sequence to be inserted, the donor usually adds selection cassettes, such as resistance genes, for the selection of engineered BAC clones [60,61]. Screening with the phenotype of the relevant marker gene considerably minimizes the background of the cells that have not successfully undergone recombination, which results in an increase in screening efficiency of more than 95% [61].

Nonetheless, there are still a number of challenges in the implementation of the single selection cassette technique. On the one hand, the insertion of a marker gene to a target gene may disrupt the function of genes that are located both upstream and downstream of the targeting site. On the other hand, even if the introduction of selection cassettes does not affect the function of the neighboring genes, when the genome needs to be continuously modified, the stacking of multiple selection cassettes in the same genome makes the screening of subsequent recombinant mutants increasingly tricky. Therefore, it is essential to eliminate screening markers.

### 3.2. Selection Cassette in Combination with Site-Specific Recombinase Recognition Motif

Site-specific recombinase systems, such as the *P1 phage*-derived Cre-*loxP* system (optimum temperature (37 °C) and the *yeast*-derived FLP-*FRT* system (optimum temperature 30 °C) [62], are also commonly used for editing herpesvirus genomes [25,63,64,65]. In 2007, an improved FLP recombinase (FLPo) was developed that works efficiently at 37 °C [66,67] and has been widely used in generating various genetically modified animal models [67,68], but it has not yet been employed in bacterial gene manipulation. As part of the editing procedure, the donor needs to add codirectional *LoxP*/*FRT* sites on both sides of the selection cassette based on the single selection cassette design. The expression of recombinase enzymes allows for the removal of the selection cassette that is located in the middle of the two sites [62].Although successful in removing the long selection cassette with an efficiency approaching 100% [69,70], the use of this technique leaves a single residual *LoxP* or *FRT* site at the intergenic site of BAC insertion and excision. It is impossible to dismiss the possibility that this residual *LoxP* or *FRT* piece will impact the transcription, translation and the function of genes located both upstream and downstream in the genome. Additionally, the accumulation of multiple *LoxP* or *FRT* sequences in the genome can interfere with the continuous editing process. As a result, researchers are actively searching for ways to perform “scarless” editing, which does not leave any trace of the editing process on the genome.

### 3.3. Selection Cassette in Combination with I-sceI Endonuclease Recognition Site (En Passant Mutagenesis)

The endonuclease I-SceI, which is derived from *Saccharomyces cerevisiae*, is a useful tool that enables scarless editing [71,72]. This tool has been extensively utilized for scarless editing of the herpesvirus genome [73,74,75].

In the first round of recombination, the donor must add a single I-SceI recognition site sequence and sequence duplications, along with the selection cassette and the homologous arm on both sides. In the second round of recombination, the I-SceI site is cleaved by the expression of the I-sceI enzyme, where the sequence duplications mediate the recombination, thus achieving scarless editing. The technology referred to as “*en passant mutagenesis*” has been shown to be effective in inducing large-scale changes in viral genomes without scarring [76] and for removing F-derivative sequences from the viral genome during reconstruction in cells [77]. However, the accumulation of point mutations or deletions within the I-SceI recognition site during the second round of homologous recombination [78,79], as well as the self-ligation of the I-SceI enzyme cleavage site, can lead to high background noise [79,80], negatively impacting screening efficiency (after the second round of recombination, the screening efficiency drops from 95% to 1–15%) [81].

### 3.4. Positive and Negative (Dual) Selection Cassettes

Applying a positive and negative (dual) selection cassette can also achieve scarless modification of the herpesvirus genome. Positive selection cassettes typically contain resistance genes, and negative selection cassettes are designed to selectively eliminate undesired modifications. In contrast, dual-selection markers use a single gene to mediate positive and negative selection under distinct conditions, achieving scarless modifications of the genome.

The *galK* gene is a dual-selection cassette widely used in genome editing of herpesviruses [82,83,84]. The *galK* gene encodes galactokinase, which allows *galK*-deficient *E. coli* to grow on the medium with galactose as the sole carbon source during positive selection. In negative selection, galactokinase can catalyze the phosphorylation of 2-deoxy-D-galactose (DOG) to produce hazardous compounds in cells. Therefore, only strains with *galK* gene deletions can grow on the DOG-containing medium [79]. Since *galK* can be utilized for both positive and negative selection in practical applications, the possibility of DOG resistance due to mutation of the *galK* gene is eliminated during positive selection [79]. Even with a relatively short homologous arm (33 bp), *galK*-mediated negative screening can still achieve an efficacy of 20–50% [79]. However, compared to resistant markers such as the kanamycin resistance gene (kan*^R^*; hereinafter designated kana), the positive selection using the *galK* gene is less efficient. In addition, basal medium, such as M63 was utilized in the *galK*-mediated positive selection process to eliminate the interference of other carbon sources. However, the bacteria grow slower in basal medium. Thus, *galK*-mediated positive selection was more time-consuming [85].

The *rpsL*-kana cassette is a positive and negative selection cassette, which is widely used in the genome editing of herpesviruses [2,86,87,88,89]. Kana mediated kanamycin resistance was utilized to produce positive selection, and this process is usually highly efficient. In negative selection, the medium containing a high concentration of streptomycin was used. The bacteria harboring the BAC with the streptomycin-sensitive *rpsL* gene (hereinafter designated *rpsL*), exhibited slower growth rate compared to the bacteria harboring the BAC without the *rpsL* [85]. However, in the actual application process, the *rpsL* gene could undergo a spontaneous mutation, resulting in resistance to streptomycin and thus increasing the background and decreasing the screening efficiency [85]. In addition, the use of *rpsL*-mediated negative selection is based on the fact that *E. coli* has a streptomycin-resistant phenotype, which limits the gene’s application. We have tried to use the streptomycin-resistant strain DH10B with the *rpsL* gene for negative screening. Unfortunately, we found that the DH10B strains of different manufacturers exhibited distinct levels of streptomycin resistance. For streptomycin sensitivity testing, multiple control groups are required to determine the optimal selection concentration. At the same time, the streptomycin sensitivity of the bacteria is greatly affected by the culture time and the concentration of the bacterial solution during the negative screening process, coupled with the extremely high background, which has a significant impact on the efficiency of negative selection.

In practical application, the negative selection efficiency mediated by the *galK* gene (16%) was significantly higher than that mediated by the *rpsL* gene (7.8%) [85]. By combining the *galK* gene with the kanamycin resistance gene, researchers have overcome the restrictions of using a single *galK* gene or *rpsL*-kana gene for herpesvirus genome editing [90,91,92]. After recombination, the positive screening process only takes 10–12 h on the kanamycin-containing plate, which improves the selection efficiency and shortens the selection cycle markedly. Based on the greater selection efficiency of the *galK* gene in negative screening compared to that of the *rpsL* gene, the *galK*-kana gene is expected to replace the *rpsL*-kana gene as an excellent screening method in scarless editing of the herpes virus. It should be noted that the negative selection cassettes can only work in specific strains of *E. coli* (Table 2).

In addition, novel selection cassettes that have emerged in bacterial genome editing but have not been applied to BACs may also provide insights for future BAC editing. In 2022, Bayer et al. modified the existing dual selection cassette *tetA* (named the *tetA^OPT^* gene) [93], which significantly increased the efficiency of both positive and negative screening to above 90% in *E. coli.* The stringently inducible toxin genes, such as the *relE* and *tse2* genes [94], mediated more efficient negative selection. These screening cassettes can be utilized in various *E. coli* strains without specific requirements and are expected to increase in the efficiency of BAC-based gene editing.

## 4. Application of BAC-Based Gene Editing in Herpesvirus Research

### 4.1. Gene Function of Herpesvirus

Since Messerle et al. [10] constructed the first viral BAC, the murine cytomegalovirus mCMV. BAC technology has definitely advanced our knowledge of the life cycle of large DNA and RNA viruses. Sleman et al. [95] constructed a double-knockout recombinant virus of human cytomegalovirus (HCMV) *UL24* and *UL43* based on the λ-red technique combined with the kana-I-sceI screening method. They demonstrated the potential role of *UL24* and *UL43* in regulating the host cell environment to facilitate immune escape by regulating the expression of downstream *UL16* binding protein 1 (*ULBP1*). Neuhierl et al. [96] constructed an EBV-BMRF1 knockout virus by combining the λ-red technique and the kana selection method. They provided solid evidence that BMRF1 is absolutely required for both DNA lytic replication and lytic protein synthesis and is also essential for the production of infectious progeny viruses of herpesviruses. This study is significant in terms of understanding the function of essential genes involved in the process of producing herpesvirus progeny. The use of BAC-based gene editing technology, which does not rely on progeny virus production, exhibits unique advantages compared to editing methods performed in eukaryotic cells. Using a similar approach, numerous researchers have successively constructed herpesvirus mutants with gene deletions such as HSV-1-*UL39* [97] and EBV-*Cp* [89], thereby studying the role of different genes on virus growth and replication, pathogenicity, and the interrelationship between the virus and the host, which has contributed to the understanding of the virus at the molecular level. At the same time, novel pharmacological targets or critical virulence factors can be found by studying unreported gene functions, which provides new ideas for developing targeted drugs and constructing attenuated vaccine vectors.

### 4.2. Gene Therapy Vectors and Vaccine Vectors

The herpesvirus genome has numerous non-essential genes that can be replaced or substituted with exogenous sequences, allowing for its modification as a gene therapy or vaccination vector. Herpesviruses are latent in the body, thus preventing them from being recognized and cleared by the immune system [98]. In addition, the latent herpesvirus genome does not integrate into the host genomic DNA but acts as an episome, thus avoiding the risk of insertional mutagenesis of the host genome [99]. Simultaneously, as glycoproteins, such as gC, gB, and gD, are critical for the recognition of the cell surface receptors and cell entry, the efficiency of targeting tumor cells can be enhanced by modifying the herpesvirus envelope glycoproteins [98]. The BAC system also provides an excellent technical platform for convenient and safe gene editing of herpesviruses, thereby facilitating the development and application of herpesvirus genomes as gene therapy and vaccine vectors.

Takahashi’s group developed the live-attenuated vOka strain of varicella-zoster virus (VZV) in the 1970s by serially passaging the wild-type parent Oka (pOka) strain of VZV in human and guinea pig cell lines. The vOka strain has been demonstrated to be effective in reducing morbidity and mortality associated with varicella [100,101]. However, vOka retains its complete neurovirulence and is capable of establishing latency and reactivating to produce herpes zoster [102] and neurological sequelae such as meningitis [103] in vaccine recipients, leading to safety concerns. Notably, the ORF7 gene is the only known full-length VZV gene that is required for virulence in both human skin and neural cells [104], while it is non-essential for viral replication in different cell cultures [105]. Wang et al. [82] constructed the pOka-derived live-attenuated varicella vaccine strain v7D in which the ORF7 gene was deleted using the λ-red recombination and *galK* dual-selection. ORF7 knockout reduced the risk of skin and nervous system complications associated with the vOka vaccine while maintaining immunogenicity [103,106,107]. v7D has become a promising and safer candidate for the live-attenuated varicella vaccine [108]. With increased safety, v7D could also be a promising delivery vector for gene therapy.

The large subunit of ribonucleotide reductase (ICP6) encoded by the *UL39* gene of herpes simplex virus type 1 (HSV-1) is required for efficient viral DNA synthesis. Studies have shown that homologs of the protein are present in cells and are usually elevated in dividing tumor cells but expressed at low levels in normal cells [109]. Thus, HSV-1 mutants with the deletion of the *UL39* gene, such as *hrR3*, can be selectively replicated in tumor cells [110]. Miao et al. [111] created YE-PC8 by replacing *UL39* of oncolytic HSV-1 with a cell cycle-adjustable luciferase transgene cassette via λ-red recombination and *galK* dual-selection technology The anti-tumor effects of YE-PC8 have been demonstrated in mouse models of hepatocellular carcinoma and subcutaneous xenografts of human gliomas, and it may be possible to develop appropriate therapeutic genes for cancer treatment further.

### 4.3. Visualization of Herpes Virus

With the development of herpesvirus genome editing technology, virus visualization research has become convenient and fast. Neubauer et al. [112] added green fluorescent protein to equine herpesvirus type 1 (EHV-1) glycoprotein K (gK) and *UL34* protein, which can detect viral protein expression in the viral life cycle. Furthermore, a recombinant murine gamma-herpesvirus-68 (MHV-68) expressing a firefly luciferase gene (Fluc) was constructed as a model representative of humanγ-herpesviruses to investigate the mechanisms of latency and reactivation of MHV. The infection of mice with the Fluc-tagged MHV-68 enabled the identification of the initial sites of lytic replication. Additionally, following the resolution of the acute phase of infection, real-time reactivation of the latent virus was demonstrated through the administration of either a proteasome inhibitor (Velcade) or an immunosuppressant (cyclosporine A) [113]. Recent research findings have also indicated that MHV-68 reactivation occurs as a result of an acute infection with parasitic worms and that the reactivation of herpesviruses is associated with specific cytokines [114].

## 5. Conclusions and Prospects

The utilization of BAC recombinant technology has significantly expanded the scope of herpesvirus research. Several systems are now available, and the technology is widely adopted among research laboratories. Nevertheless, creating recombinant viruses through this technique can be laborious and time-consuming. There is still a need for improvement in the efficiency of BAC-based gene editing. Establishing more effective BAC-based viral genome editing technology will increase the precision and efficiency of genome editing while decreasing the time and resources necessary for testing.

The advancements in BAC-based genome editing techniques will play a vital role in facilitating the study of herpesvirus gene function and vaccine development, ultimately advancing our understanding of herpesvirus infections and the development of more effective vaccines and drugs for the prevention and treatment of herpesviruses.

## Figures and Tables

**Table 1:** The pros and cons of BAC-based herpesvirus genome editing techniques.

Editing Techniques	Length of Homologous Arms	Editing Efficiency	Requirement for Editing Site	Stability of BAC
RecA Recombination	500 bp–3 k bp	10^−6^ to 10^−4^	None	Causes mutations in the BAC
λ-red Recombination	30–50 bp	<1%	None	Maintained the stability of BAC
Base Editing	Not Required	Approaching 100%	PAM Sites & Base Editing Sites; Only Base Editing can be done	Maintained the stability of BAC

**Table 2:** The pros and cons of screening methods for herpesvirus mutants.

Screening Methods	Feasibility of Continuous Editing	Applicable *E. coli* Strain Type	Selection Efficiency	Auxiliary Proteins	Feasibility of Scarless Editing	Duration of Each Editing Cycle
Single selection	Difficult	All	>95%	None	No	3 Days
combination site-specific recombinase recognition motif	Difficult	All	Approaching 100%	FLP/Cre	No	7 Days
Combination I-sceI endonuclease recognition site	Feasible	All	Negtive: 1–15%	I-sceI	Yes	7 Days
Positive and negative (dual) selection cassettes	Feasible	*rpsL*; Str^R^ *galK*; Δ*galK*	Negative: *rpsL*-7.8% *galK*-16%	None	Yes	*rpsL*-Kana: 7 Days *galK*: 12 Days *galK*-Kana: 9 Days
